# Evaluating the performance of multilingual models in answer extraction and question generation

**DOI:** 10.1038/s41598-024-66472-5

**Published:** 2024-07-05

**Authors:** Antonio Moreno-Cediel, Jesus-Angel del-Hoyo-Gabaldon, Eva Garcia-Lopez, Antonio Garcia-Cabot, David de-Fitero-Dominguez

**Affiliations:** Departamento de Ciencias de la Computación, Universidad de Alcalá, 28805 Alcalá de Henares, Spain

**Keywords:** Computer science, Software

## Abstract

Multiple-choice test generation is one of the most complex NLP problems, especially in languages other than English, where there is a lack of prior research. After a review of the literature, it has been verified that some methods like the usage of rule-based systems or primitive neural networks have led to the application of a recent architecture, the Transformer architecture, in the tasks of Answer Extraction (AE) and Question Generation (QG). Thereby, this study is centred in searching and developing better models for the AE and QG tasks in Spanish, using an answer-aware methodology. For this purpose, three multilingual models (mT5-base, mT0-base and BLOOMZ-560 M) have been fine-tuned using three different datasets: a translation to Spanish of the SQuAD dataset; SQAC, which is a dataset in Spanish; and their union (SQuAD + SQAC), which shows slightly better results. Regarding the models, the performance of mT5-base has been compared with that found in two newer models, mT0-base and BLOOMZ-560 M. These models were fine-tuned for multiple tasks in literature, including AE and QG, but, in general, the best results are obtained from the mT5 models trained in our study with the SQuAD + SQAC dataset. Nonetheless, some other good results are obtained from mT5 models trained only with the SQAC dataset. For their evaluation, the widely used BLEU1-4, METEOR and ROUGE-L metrics have been obtained, where mT5 outperforms some similar research works. Besides, CIDEr, SARI, GLEU, WER and the cosine similarity metrics have been calculated to present a benchmark within the AE and QG problems for future work.

## Introduction

### Motivation

The Question Generation (QG) task is one of the main Natural Language Processing (NLP) problems, and it is defined as the automatic generation of questions from inputs such as text, raw data and knowledge bases^1^. Specifically, an answer-aware Question Generation system performs the QG task given a target answer (right answer) as an input, besides the passage. Therefore, the task of Answer Extraction (AE) is essential for this system, and it is defined as the task of obtaining a fragment representing a plausible answer directly from the input passage, with no given question.

Answer-aware QG systems can be applied in several real applications such as the creation of open-domain chatbots^2^, the improvement of Question Answering (QA) systems^3^ or the creation of new automatic evaluation metrics^4^. Additionally, QG contributes to the appearance of newer fact verification techniques^5^ and to the automatization of some educational issues. Particularly, in the field of education, Le et al.^6^ stated that it can be used for knowledge or skills acquisition (e.g. writing skills^7^), as well as for knowledge assessment (e.g. multiple choice questions^8,9^ and the creation of tutorial dialogues^10^).

Even though recent advances in NLP achieved extraordinary results in QG systems, NLP applications are hugely language dependent. In this sense, research in languages other than English is very scarce, i.e., there are much fewer resources available. Particularly, in Spanish there are not enough native models and datasets to perform greater investigations on the AE and QG fields with promising results. Consequently, trying to solve the English language dependency in the field of NLP, some multilingual models have been proposed. These models are pre-trained in several languages and are able to work reasonably well in languages other than English.

### Contribution

This research focuses on the evaluation of the performance of three multilingual models (mT5, mT0 and BLOOMZ) in the AE and QG tasks using two datasets in Spanish (SQuAD, SQAC) and their union. This evaluation has been made using several automatic evaluation metrics such as BLEU1-4, ROUGE-L, METEOR, etc. In addition, less common metrics like SARI, GLEU or WER have been calculated. As a result, the metrics calculated allows the comparison of this work with previous research and future work in the AE and QG fields, especially in Spanish. This is important, especially regarding the AE task, because no previous benchmarks have been found for this task, not only in Spanish, but not in English either.

Our study applies the previous research done in English to Spanish, a language with fewer resources available, testing different models and datasets, establishing a way forward, achieving relevant results and providing a benchmark to guide and compare future works.

In summary, the main contributions of this research are: (1) The extensive adaptation of three different multilingual models (mT5, mT0 and BLOOMZ), specifically tuned to the nuances of the Spanish language, to increase their efficiency in AE and QG tasks; (2) A meticulous evaluation and comparison of the three multilingual models, focusing on their respective performance when fine-tuned on individual (SQuAD, SQAC) and combined Spanish datasets, offering deeper insights into their adaptability and efficacy; (3) An in-depth study of the inherent abilities of the mT0 and BLOOMZ models in a zero-shot setting, comparing their generalization capabilities with scenarios in which they are fine-tuned to the Spanish language; and (4) The establishment of a robust AE and QG benchmark, based on extensive metric evaluations, both common and less used, setting a standard that not only encapsulates the progress made in the field, but also provides a direction for future research efforts in AE and QG, especially for the Spanish language.

### Article organization

The rest of this paper is divided into four parts. Section II summarizes the state of the art around the QG and AE tasks. Section III, Materials and methods, explains the different datasets, models, data preprocessing techniques and automatic evaluation metrics that have been used. Next, the results obtained are stated in Section IV. Finally, in Section V, we discuss and compare the results obtained with the state-of-the-art and provide a conclusion of our study.

## Related work

During the last decades, wide ranges of approaches have been used to solve the Question Generation (QG) problem. Firstly, rule-based systems were used^8,11,12^. However, they require deep knowledge of linguistics to construct well designed rules capable of transforming declarative sentences into interrogative ones. Additionally, this approach requires high maintenance, being necessary to add or modify new rules if a new type of question is needed. Afterwards, Heilman and Smith^13^ proposed a solution that generates multiple questions for a given context and later ranks each question with a logistic regression model. As a result, more recent studies make use of attention based neural models for QG, thus outperforming previous rule-based systems^14^. Finally, up to date research is trying to stand out the power of attention of the models avoiding the issue of generating irrelevant and uninformative questions. This objective is mainly achieved with Transformer based models, which point out the most relevant fragments of the passage^15,16^. Specifically, Chan and Fan^17^ investigate the use of the BERT model for the QG task, finding out a simple but effective input encoding scheme with highlighting tokens. Furthermore, other strategies, such as the question generation from facts^18^ or the incorporation of answer information (answer-aware QG)^19–22^, have been proposed to tackle this problem. According to these studies, answer-aware QG outperforms previous results. Consequently, with this strategy, the schema changed, making necessary the acquisition of answers directly from the passage without a given question. Based on this technique, Liu et al.^23^ generate questions including a clue word predictor, to identify a word or phrase that is helpful to guide the way to ask a question, and a Graph Convolutional Network-based clue predictor, to learn whether each word in source text can be copied to the target question or must be generated. Moreover, Sasazawa et al.^24^ generate questions incorporating an interrogative phrase at the end of the passage, trying to avoid the generation of inappropriate questions whose interrogative phrases do not match the target answers. However, Kim et al.^25^ expose that, in answer-aware QG, a significant proportion of the generated questions tend to include words of the answer, resulting in the generation of improper questions. Therefore, they propose an answer separation technique to address this issue. Even though the results were better, newer studies stand out the problem of including in the question wrong keywords and question words and propose a neural answer-aware QG model with sentence-level semantic matching, answer position inferring and gated fusion^26^.

However, even though neural models outperform previous rule-based strategies^14^, they need huge amounts of data providing only one question per inference. This problem has been defined by A. Naeiji et al.^27^ as the single-question-generation-problem. To tackle this issue, a hybrid approach is proposed, combining a sequence-to-sequence model with techniques used in rule-based systems. As a result, this research can generate multiple and diverse questions without specifying the number of questions to be generated or requiring keyword-labeled training data, thus obtaining relevant progress in this field.

Besides, another challenge regarding the QG task is the correlation between question types and their corresponding answers. Subsequently, current research supports the idea of including a classifier to improve the performance of the question generator model. More specifically, Sun et al.^28^ improved the accuracy of interrogative words in generated questions by using a question type classifier based on a pretrained language model and a question generator. The question type classifier is constructed based on BERT^29^ and fine-tuned with nine question categories, while the question generator model is based on the model proposed by Sun et al.^30^.

Regarding the Answer Extraction (AE) problem, most of the approaches obtain the answer given a passage and the corresponding question (Question Answering) instead of obtaining the answer directly from the text (AE). This methodology was firstly accomplished with rule-based methods^31^ and more recently with Transformer-based neural networks^32,33^. Even though the Question Answering technique is the most popular, other studies apply the idea of extracting answers directly from the passage, without a given question. This issue has been addressed by using a wide range of techniques. Firstly, linguistic tags and rules were used^34^. Secondly, See et al.^35^ proposed an attention-based pointer generator model, created as a hybrid between a pointer network^36^ and a sequence-to-sequence model with attention (similar to the one proposed by Nallapati et al.^37^). Then, Subramanian et al.^38^ made use, exclusively, of pointer networks trained to point sequentially to the start and end locations of the answers. This approach was then improved by Sun et al.^39^, using feature-enriched pointer generator models, by adding features such as named entity (NE) or part-of-speech (POS) in the embedding layer of the encoder. Later, a BERT^29^ encoder was used to predict answer spans from the passage. Once several answers have been obtained, they are sorted according to a confidence score and the best answers are selected^40^. In addition, the research done by Rodriguez-Torrealba et al.^41^ use fine-tuned T5 models to create multiple-choice questions following the answer-aware technique. Moreover, the use of the Stanford CoreNLP tagger^42^ is another common methodology to address the AE task^43^. Using the mentioned tagger, Arumae and Liu^44^ applied summarization techniques to improve the AE process performance. Additionally, Dugan et al.^45^ used a T5 language model^46^ and proved that providing human-written summaries, or automatically generated ones (instead of the source passage), reinforces AE and QA tasks.

Finally, Uto et al.^47^ have performed a research study about the difficulty control of QG. For this purpose, a hybrid strategy using BERT^29^ for AE and GPT-2^48^ to perform answer-aware QG is proposed. Additionally, the difficulty control problem is addressed using the item response theory (IRT)^49^, making it possible to select the difficulty of the generated question–answer pairs.

The evaluation of these studies must be captured with some metrics to be able to compare the performance within different strategies. Numerous research studies make use of the BLEU 1–4 metrics^50^ to evaluate the quality of machine-translated text. Additionally, the METEOR metric^51^ is also used, since it addresses some weaknesses of BLEU (e.g. including recall). Moreover, ROUGE-L^52^ is employed to measure the longest common subsequence between two sequences of characters. As a result, recent studies have published benchmarks including these metrics^53^. However, these metrics are not specific for the QG or AE problems. They are borrowed from other natural language generation (NLG) tasks, where they have shown to correlate weakly with human opinion^54^. Laban et al.^55^ ratify this idea by using a new evaluation technique for QG in which a teacher selects a quiz concept, chooses which candidate questions from various models to include in the quiz and gives a reason to reject the others, concluding that better metrics are needed to guide future progress in QG and NLG research.

## Materials and methods

This work follows an answer-aware QG technique. Since the use of this technique requires to previously know the answer, it will be obtained in advance by following an AE methodology. Both the AE and QG tasks have been addressed with three different models: mT5-base, mT0-base and BLOOMZ-560 M, by generating question–answer pairs. Finally, these pairs are evaluated by using different metrics, making possible the performance comparison of the models in the AE and QG problems.

### Datasets

Regarding data, two datasets are being used, SQAC and SQuAD. Additionally, a new one was created by joining the SQuAD and SQAC datasets. These datasets are explained below.

SQAC: the Spanish Question Answering Corpus (SQAC) is a set of 18,817 answerable questions mainly extracted from the Spanish Wikipedia and the Spanish Wikinews websites^56^.

The dataset was created using an extractive method, so additional knowledge is not required to answer those questions, as they are based on their associated texts.

SQAC contains 15,036 examples in the training set, and its validation and test sets have been joined into a single validation set that contains a total of 3,774 rows.

SQuAD: the Standford Question Answering Dataset (SQuAD) is a selection of questions based on a collection of articles obtained from the English Wikipedia^57^.

It was created due to the need for a large and high-quality dataset. In this study, the first version (v1.1) is used, as every question has its answer, as opposed to the second version (v2.0), which includes 50,000 + unanswerable questions.

Since our objective is to create Spanish-based models, we used an automatic translation of this dataset.

It contains 87,595 examples in the training set and 10,570 in the validation set.

SQAC + SQuAD: this dataset is the union of the two previous ones. This combination was made to evaluate if models trained with this data can generalize better. Likewise, we assess if the generated predictions are written in a better Spanish, as SQAC was created in this language from scratch. Finally, since deep learning methods need as much information as possible to train properly, this experiment is performed to analyze whether the improvements are substantial or not.

The resulting dataset is composed of 102,631 questions on the training set, being 15,036 examples from the SQAC dataset and 87,595 from SQuAD. Besides, their validation sets have been gathered into a 14,344-example validation set, so the performance of each model can be computed globally for the union of both datasets.

### Models

We are using two similar encoder-decoder models (mT5-base and mT0-base) and one only-decoder model (BLOOMZ-560 M). All these are multilingual models, pre-trained with several languages, including Spanish.

These models were included into a pipeline that generates multiple-choice tests in Spanish, hence we look for different ways to improve the quality of questions and answers. Thus, our research study focuses on the comparison between different approaches.

Firstly, we used mT5 since text-to-text models are suitable for generative tasks. These models receive some text as an input, then they return another text as an output, which is, in our case, an answer (AE) or a question (QG).

mT5 is a multilingual variant of T5 pre-trained with mC4, a version of the C4 dataset with examples in 101 different languages^58^. mT5 inherits T5 properties, which has some implications for the inputs, since the task needs to be specified for the model to give an appropriate output^46^. The architecture of mT5, along with mT0, is based on that of T5, specifically the enhanced T5.1.1 version. This updated version improves upon the original T5 by incorporating features such as GeGLU nonlinearities^59^.

Once we had a benchmark to compare, we also fine-tuned mT0 and BLOOMZ, which are multitask fine-tuned versions of the original mT5 and BLOOM models, respectively, optimized for enhanced crosslingual and multitask capabilities. Notably, BLOOMZ adopts a decoder-only architecture and integrates modern enhancements, like ALiBi Positional Embeddings^60^ and an additional post-embedding normalization layer for better training stability. Due to the token limit that affects BLOOMZ, which can only process up to 2,048 tokens^61^, and due to hardware limitations, the data preprocessing was adapted to tackle these issues.

### Data preprocessing

Since two different problems are solved, a separate format is used for AE and QG. Nonetheless, processing is common to both of them.

Due to the token limitation mentioned above, we needed to reduce the input length below 2,048 tokens for some contexts. However, because of further hardware limitations, the inputs were finally limited to 1,024 tokens for the mT0 and BLOOMZ models, and 512 tokens for the mT5 model. The context length, in addition to some relevant hyperparameters used during the training of the models, can be found in Table 1. While removing tokens, the part of the context related to the target (the desired output) could be deleted. To solve this, we highlight either the phrase where the answer is (AE) or the answer from which the question will be generated (QG) with the tag < h1 > , so the parts of the context that do not contain that label can be removed.

**Table 1:** Hyperparameters used to train the mT5, mT0 and BLOOMZ models, respectively.

	*mT5*		*mT0*	*BloomZ*
	*QG*	*AE*		
Optimizer	AdamW	AdamW	AdamW	AdamW
Learning rate	0.00005	0.00005	0.00005	0.00005
Weight decay	0.01	0.01	0.01	0.01
Input Lenght	512	512	1024	1024
Batch size	4	2	4	4
# epochs	12	8	3	3

The mT5 model was trained with different hyperparameters for its QG and AE versions due to hardware limitations, while mT0 and BLOOMZ were trained using the same hyperparameters in both cases.

This technique is called context-aware answer extraction/question generation, and helps to reduce the number of tokens. In addition, it makes models focus on a limited span of context. In this case, the mentioned span would contain the targeted answer or a phrase to extract answers from. This provides more accurate results because the models will not be centered on other possible occurrences of the answers throughout the input^62^.

Please note that a similar data preprocessing step must be carried out before inference.

1. For the AE task, a context with *n* sentences will generate *n* inputs for the AE model. In each of those inputs, a different sentence must be highlighted with the < *h1* > tag, and this must be disjoint from the highlighted sentences in other inputs.
2. Regarding the QG task, every input must have exactly one answer highlighted within the context, using the < *h1* > tag. The answer may be extracted and labelled manually, or alternatively, an AE model may be employed to retrieve an answer to automatically label it within the context.

Regarding the tokenizer, it was trained with the special token < *sep* > , which lets the model identify different important fragments. For example, this token can separate the ending of an answer with the beginning of the next one or to split the information given to the model (passage) from the command (extract answers).

AE format: first, a feature selection is performed to obtain the title, context, and answers from each row. Then, for each context in the dataset, its answers are gathered. To group the different answers that belong to the same context, the title is used, which will be discarded after this step.

With this procedure, we have removed duplicated contexts. Now, we want to delimit every phrase of the passage, obtained by *sent_tokenize*^63^, so the answers can be grouped per phrase using their *answer_start* attribute. *sent_tokenize* retrieves the *n* sentences within a text. For each of those sentences, a duplicate of the original context is created, in which its corresponding sentence is highlighted with the < *h1* > tag. Consequently, *n* possible inputs are obtained. Then, once the answers have been gathered by the sentence they belong to, they are formatted as it follows: *answer1* < *sep* > *answer2* < *sep* > *…* < *sep* > *answerN* < *sep* > , where every answer belongs to the same sentence. Finally, *input-target* pairs (registers) are created, where the input is a context with a highlighted sentence and its target are the related answers, so the texts without a target are discarded.

**Figure 1:**
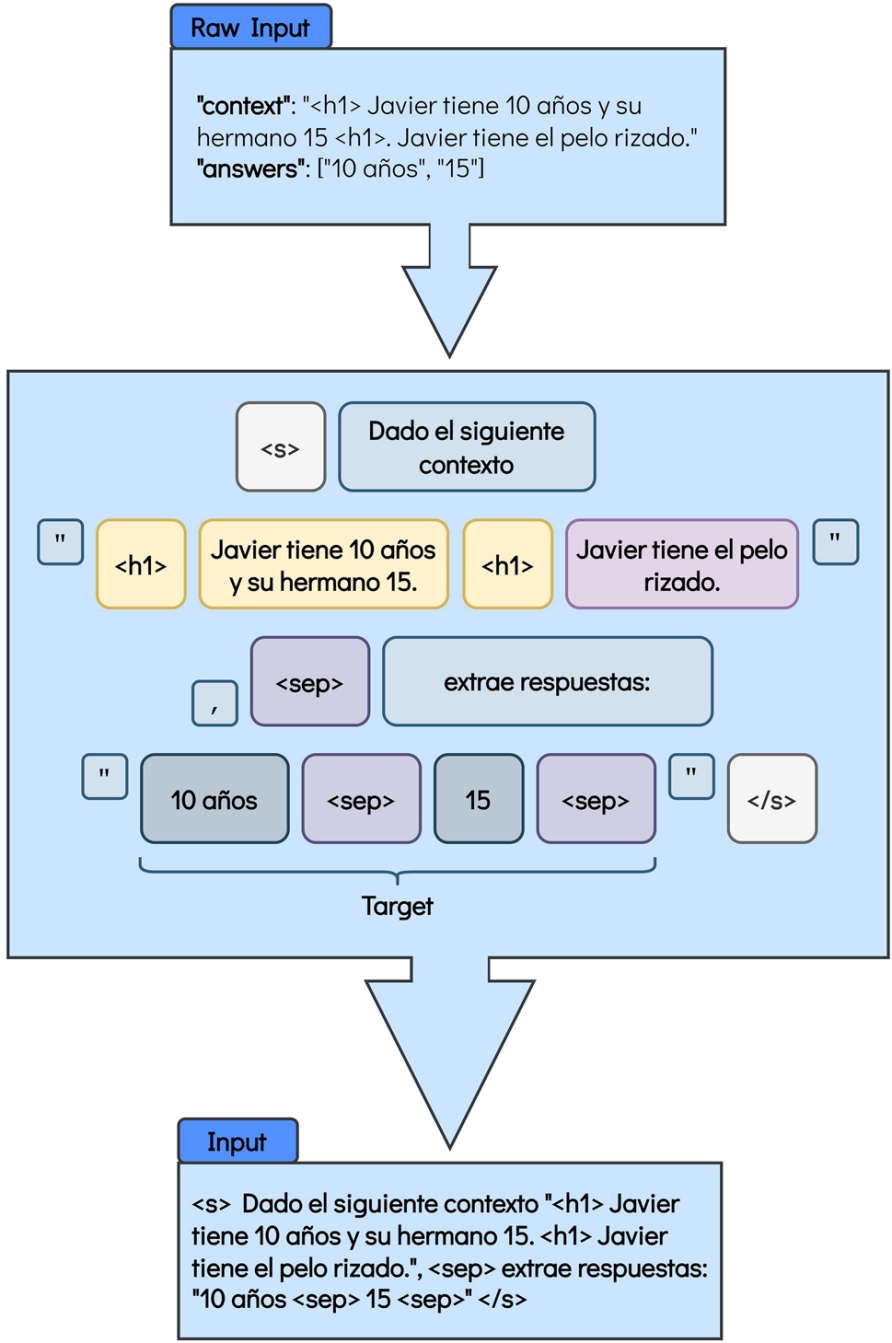
Format for the BLOOMZ model for the Answer Extraction task. The < s > token marks the beginning of the input, then the first part of the prompt is given. The part between quotation marks is the context, and the text contained between the < h1 > tag is the span where the model must focus on. Then, the second part of the prompt is given, after the < sep > token, which indicates the beginning of the target. < /s > means end of sentence. The translation into English is the following: Raw Input: “context”: “ < h1 > Javier is 10 years old and his brother is 15. < h1 > Javier has curly hair.” “answers”: [“10 years old”, “15”]. Figure’s main body: < s > Given the following context “ < h1 > Javier is 10 years old and his brother is 15. < h1 > Javier has curly hair.”, < sep > extract answers: “10 years old < sep > 15 < sep > ” < /s > . Input: < s > Given the following context “ < h1 > Javier is 10 years old and his brother is 15. < h1 > Javier has curly hair.”, < sep > extract answers: “10 years old < sep > 15 < sep > ” < /s > .

**Figure 2:**
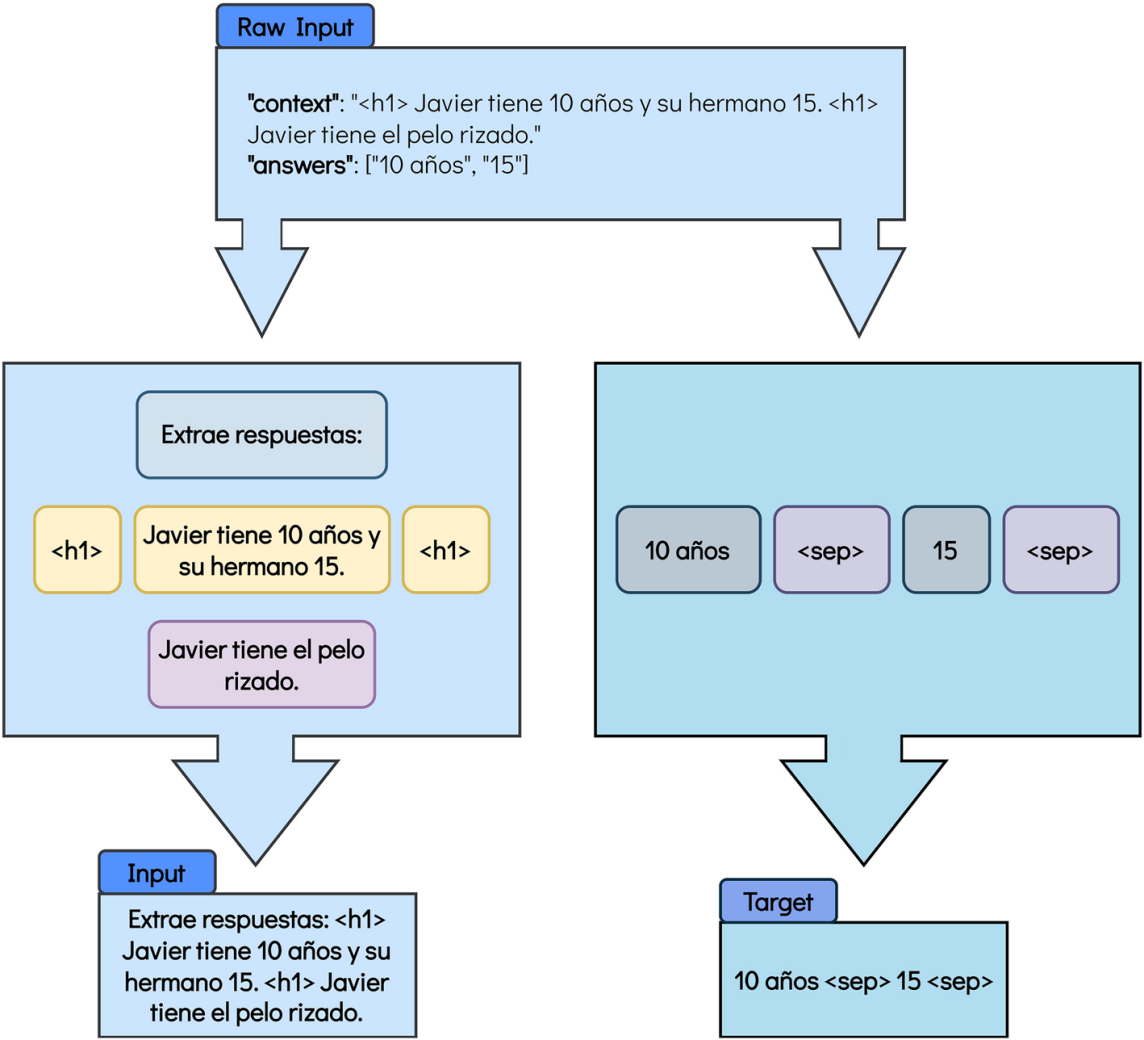
Format for the mT0 and mT5 models for the Answer Extraction task. The translation into English is the following: Raw Input: “context”: “ < h1 > Javier is 10 years old and his brother is 15. < h1 > Javier has curly hair.” “answers”: [“10 years old”, “15”]. Figure’s main body (left): Extract answers: < h1 > Javier is 10 years old and his brother is 15. < h1 > Javier has curly hair. Figure’s main body (right): 10 years old < sep > 15 < sep > . Input: Extract answers: < h1 > Javier is 10 years old and his brother is 15. < h1 > Javier has curly hair. Target: 10 years old < sep > 15 < sep > .

Last, we format the input to indicate the task they need to perform. As mT5 and mT0 are similar models, we used the same prompt for both. For BLOOMZ, we used a prompt like the suggested templates in the work of Muennighoff et al.^61^. The resulting inputs can be better seen in Figs. 1 and 2.

QG format: unlike the previous task, in the Question Generation problem only one question per answer is obtained, so removing duplicated contexts is not required.

**Figure 3:**
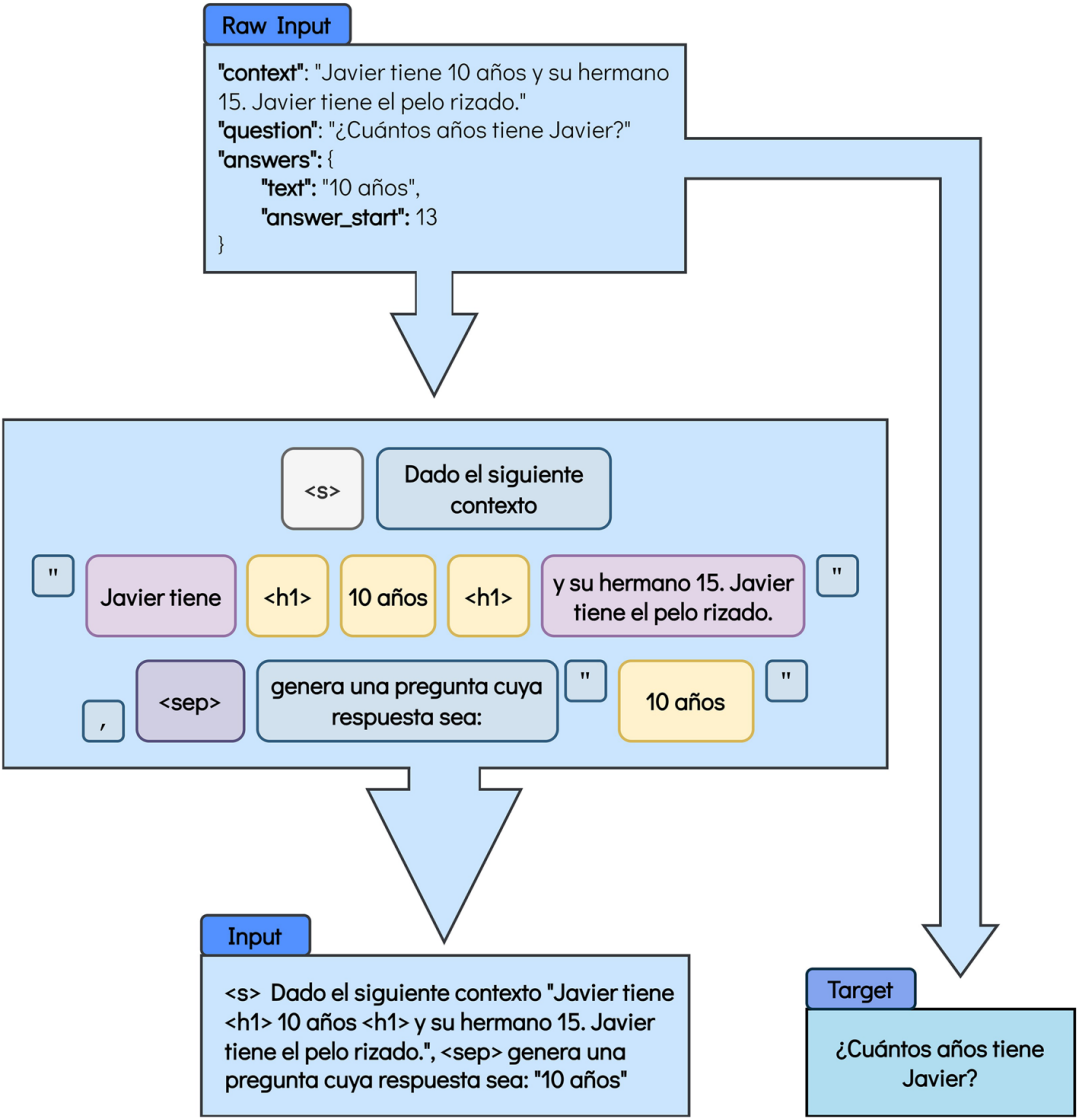
Format for the mT0 model for the Question Generation task. In this case, mT0 has a similar format to BLOOMZ. Nonetheless, as mT0 is an encoder-decoder model, the target is separated from the input. The translation into English is the following: Raw Input: “context”: “Javier is 10 years old and his brother is 15. Javier has curly hair.” “question”: “How old is Javier?” “answers”: {“text”: “10 years old”, “answer_start”: 13}. Figure’s main body: < s > Given the following context “Javier is < h1 > 10 years old < h1 > and his brother is 15. Javier has curly hair.”, < sep > generate a question whose answer would be: “10 years old”. Input: < s > Given the following context “Javier is < h1 > 10 years old < h1 > and his brother is 15. Javier has curly hair.”, < sep > generate a question whose answer would be: “10 years old”. Target: “How old is Javier?”.

**Figure 4:**
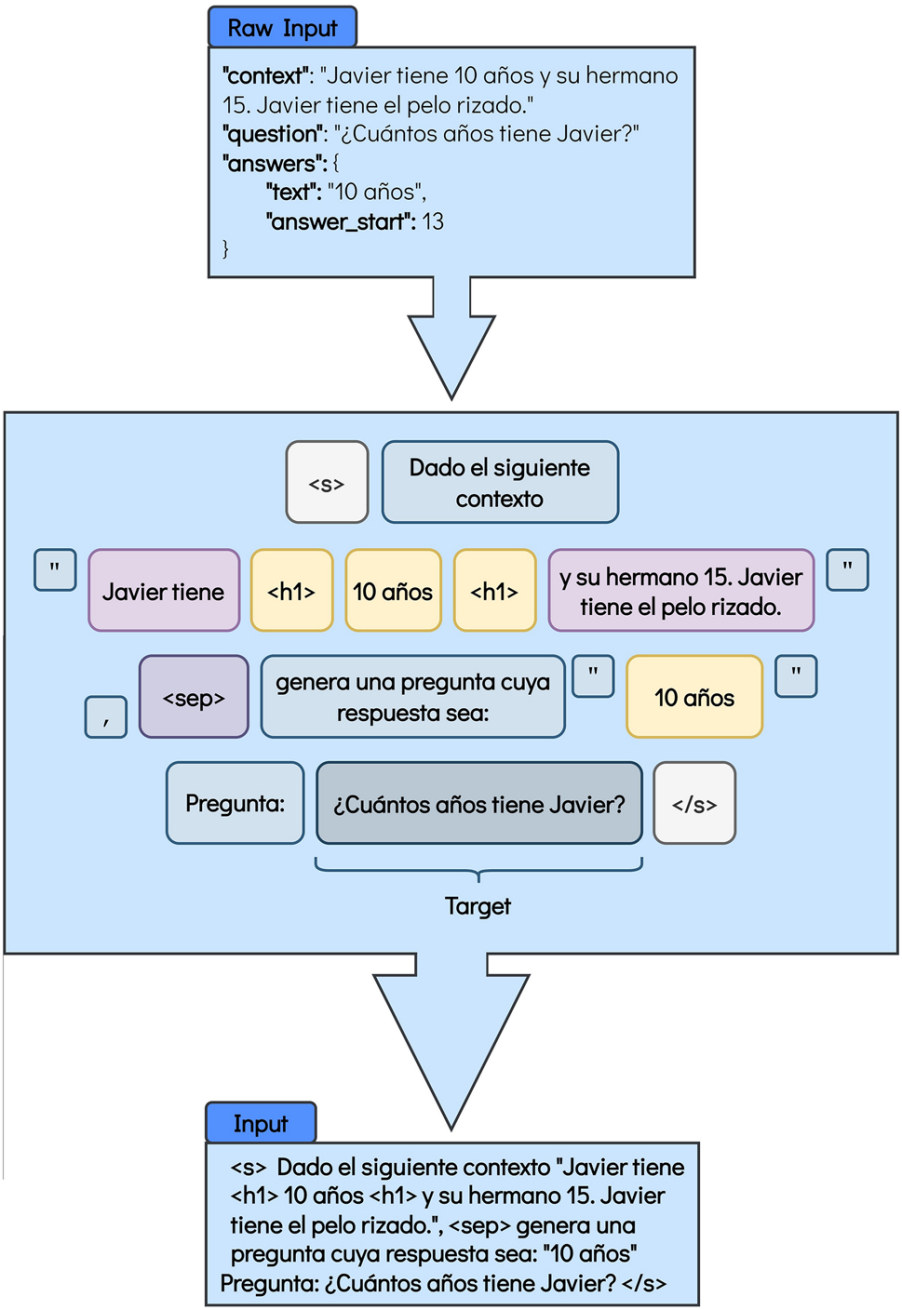
Format for the BLOOMZ model for the Question Generation task. The translation into English is the following: Raw Input: “context”: “Javier is 10 years old and his brother is 15. Javier has curly hair.” “question”: “How old is Javier?” “answers”: {“text”: “10 years old”, “answer_start”: “13”}. Figure’s main body: < s > Given the following context “Javier is < h1 > 10 years old < h1 > and his brother is 15. Javier has curly hair.”, < sep > generate a question whose answer would be: “10 years old” Question: How old is Javier? < /s > . Input: < s > Given the following context “Javier is < h1 > 10 years old < h1 > and his brother is 15. Javier has curly hair.”, < sep > generate a question whose answer would be: “10 years old” Question: How old is Javier? < /s > .

**Figure 5:**
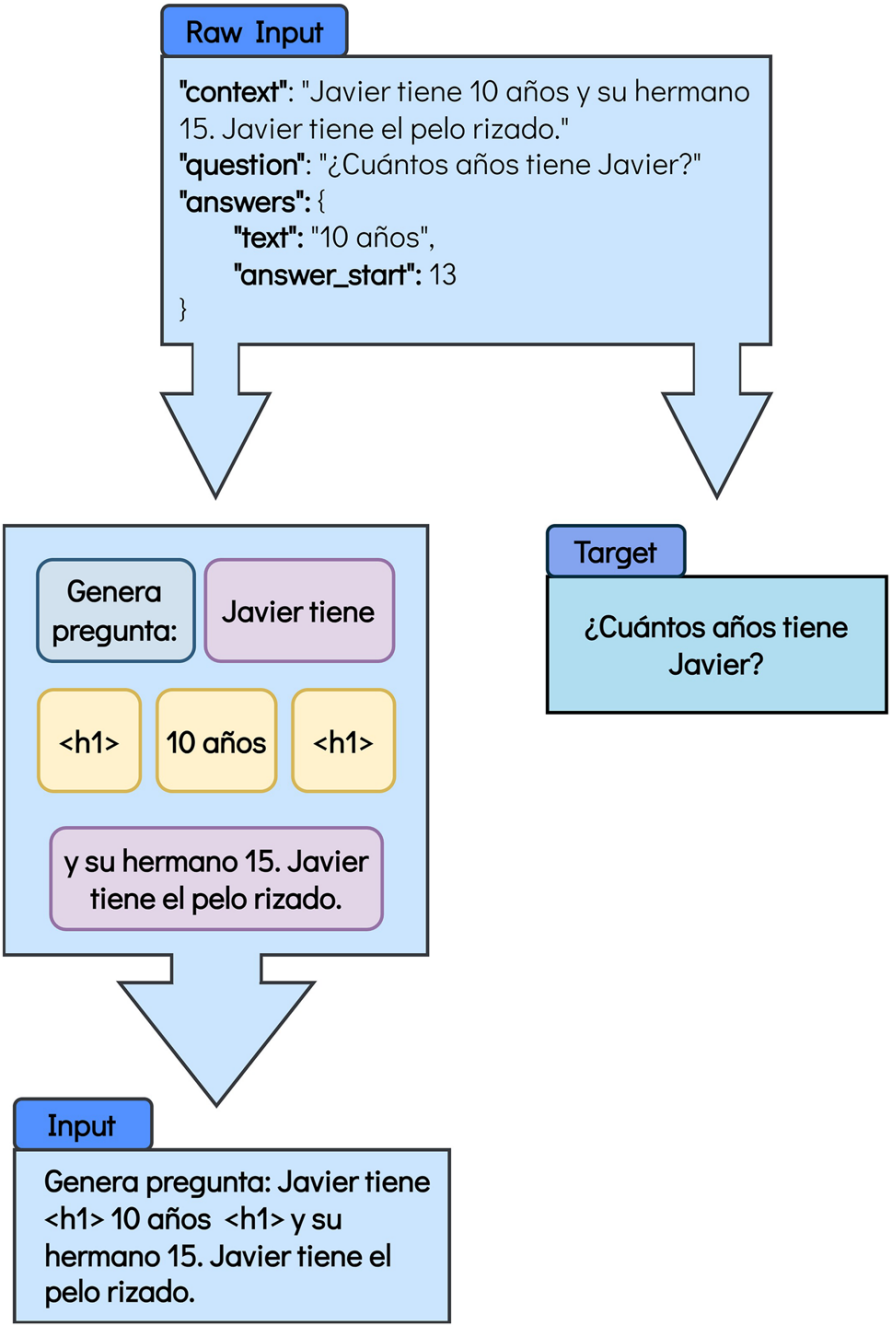
Format for the mT5 model for the Question Generation task. The translation into English is the following: Raw Input: “context”: “Javier is 10 years old and his brother is 15. Javier has curly hair.” “question”: “How old is Javier?” “answers”: {“text”: “10 years old”, “answer_start”: 13}. Figure’s main body: Generate question: Javier is < h1 > 10 years old < h1 > and his brother is 15. Javier has curly hair. Input: Generate question: Javier is < h1 > 10 years old < h1 > and his brother is 15. Javier has curly hair. Target: “How old is Javier?”.

To reduce the dimensionality of the problem, the context, the targeted question, and the answer that would respond to the question are selected. The answer is then used to highlight it in the context using the < *h1* > tag, as previously mentioned. In the case of the mT5 model, the answer is discarded, as it is no longer needed, and a simple prompt is added, as illustrated in Fig. 5. Conversely, mT0 and BLOOMZ models also require the answer for the prompt creation (Figs. 3 and 4), and thus, it is dropped after the prompt is generated and coupled to the rest of the input. In these cases, the template changes from the previous task, and it is shown in Figs. 3, 4 and 5 for the mT0, BLOOMZ and mT5 models, respectively. Please note that, same as for the QG task, a prompt similar to those suggested by Muenninghoff^61^ for the BLOOMZ and mT0 models is being used.

The algorithms used for both AE and QG can be found in Appendix A of the Supplementary Information.

### Evaluation metrics

Once the different models have been trained, inference is done with the validation set of each dataset, obtaining some predictions to compute the different metrics. These metrics have been applied to conclude which strategy provides better results for the AE and QG problems.

The BLEU 1–4^50^, METEOR^51^, ROUGE-L^52^ and CIDEr^64^ metrics are calculated by using the *nlg-eval* tool^65^. Besides, the cosine similarity obtained from the vectorial representation of the text^66^ has been calculated using the *sentence_similarity_spanish_es* model. Finally, the SARI^67^, GLEU^68^ and WER^69,70^ metrics have been obtained to study their performance in the AE and QG problems as discriminators between models, all of them computed using HugginFace’s library “Evaluate”.

- **BLEU:** this metric was proposed for evaluating machine-translated text. It compares n-grams overlapped between the original sentence and the predicted one. In this study, it is used to evaluate the quality of the generated questions and answers where it is expected that, the more similar they are to the reference, the greater fidelity they have with the original text, which is crucial for multiple-choice test generation.
- **METEOR:** this metric is used to evaluate results in machine translation problems. It performs unigram alignments between the reference and predictions, which are based on exact, stem, synonymy and paraphrase matches^65^. It solves some issues that BLEU presents (e.g., lack of recall, the use of higher order n-grams and the geometric averaging of those n-grams), so it could be a better metric to evaluate whether two questions preserve their meaning despite being paraphrased.
- **ROUGE-L:** it extracts the longest common subsequence between a given reference and the obtained prediction. Since the generated questions may differ from the original ones but can preserve their original meaning, this metric is not a good discriminator for QG models. On the contrary, in the AE problem, the main objective is to extract answers directly from the text, so rephrasing or using synonyms is not entirely correct, as the original meaning may be distorted. Thus, a higher ROUGE-L value in AE means that predictions are faithful to the original answers.
- **CIDEr:** this metric was proposed for evaluating the quality of image descriptions. In a similar way to the metrics explained above, it compares n-grams among the several descriptions given as inputs, using a stemmed representation of the text. The most frequent n-grams will be given a lower weight, as it is understood that they are likely to be less informative. Thus, CIDEr could be a good metric to evaluate whether the questions are meaningful or not.
- **Cosine similarity:** this metric is computed using a vectorial representation of the text. With the obtained vectors, the cosine of their angles can be calculated, which provides an intuition of the similarity of those two words. Please note that similar (related in meaning) words have values close to one (1), whilst identical words present a value of one (1). Similarly, the values of unrelated words are close to zero (0), and orthogonal vectors will have a value of zero (0).
- **SARI:** this metric compares the System output Against References and the Input sentence. It is computed by calculating the precision and recall for word additions, rewarding those that are found in the references and penalizing the ones that are not. Similarly, the precision and recall are calculated for those words kept in the output, and the precision for the deleted ones. In a last step, the arithmetic average is calculated, using the F1-score of the added and kept words and the precision of deletions. Xu et al.^67^ state that SARI is more correlated with simplicity than meaning, where other metrics have a better performance (e.g., BLEU). Considering both metrics (SARI and BLEU), two different models could be compared to analyze which of them generates questions that better preserve their original meanings, and then choose the model which provides simpler and more meaningful questions.
- **GLEU:** the GLEU score is a metric proposed by Wu et al.^68^ and is a slightly modified version of the BLEU metric. According to the study, since BLEU was designed as a corpus measure, its performance misbehaves when evaluating individual sentences. Hence, GLEU tries to solve this undesirable property of BLEU. The procedure is the following: first, it extracts n-grams of 1, 2, 3 or 4 tokens from the target and output sequences. Then, recall and precision are calculated and, finally, the minimum between them is chosen. Note that this metric is bounded between 0 (no matches) and 1 (exact match). In this study, since metrics are computed by comparing sentences individually, GLEU may provide better results than BLEU.
- **WER:** Word Error Rate is a commonly used metric in speech recognition problems. It considers the number of substitutions, deletions, insertions, and correct words in a predicted text, given a reference. In this study, for the AE and QG problems, WER is used to evaluate how each prediction differs from its original reference. One problem must be taken into consideration: deletions and insertions are not symmetric. This issue may make WER misbehave, giving values greater than 1^69^.

## Results

Using the evaluation metrics, several results have been obtained. Tables 2, 3 and 4 show the performance of the three models, finetuned with different datasets, on the AE task, and Tables 5, 6 and 7 on the QG task. Tables 8 and 9 present the results of performance of the mT0 and BLOOMZ base models on these tasks for the different datasets. Each table has three columns for each model: the first one shows the results obtained for the SQuAD validation set, the second one for the SQAC validation set and the third one for the SQuAD and SQAC validation sets combined together. This last column has been calculated to show the performance of the model in a more general context, with different passages and question structures from both datasets. However, it must be taken into consideration that the SQAC dataset contains more records than SQuAD.

**Table 2:** Evaluation metrics for mT5, mT0 and BLOOMZ finetuned for answer extraction with the SQuAD dataset.

*Models trained with SQuAD (AE)*									
Metric	*mT5*			*mT0*			*BloomZ*		
	SQuAD	SQAC	SQAC + SQuAD	SQuAD	SQAC	SQAC + SQuAD	SQuAD	SQAC	SQAC + SQuAD
Bleu_1	**0.4304**	**0.3528**	**0.4194**	0.3897	0.3143	0.3794	0.2794	0.1334	0.2465
Bleu_2	**0.3519**	**0.2799**	**0.3415**	0.3129	0.2481	0.3053	0.1999	0.0918	0.1758
Bleu_3	**0.3047**	**0.2463**	**0.2967**	0.2701	0.2214	0.2658	0.1661	0.0770	0.1468
Bleu_4	**0.2658**	**0.2215**	**0.2601**	0.2347	0.2028	0.2338	0.1413	0.0673	0.1257
METEOR	**0.3106**	**0.2862**	**0.3075**	0.2878	0.2714	0.2864	0.2367	0.1680	0.2228
ROUGE_L	**0.5173**	**0.4702**	**0.5102**	0.4946	0.4514	0.4887	0.3821	0.2578	0.3583
CIDEr	**2.4923**	**2.1517**	**2.4539**	2.2603	1.9623	2.2324	1.2463	0.7667	1.1478
Cos_sim	**0.7975**	**0.7392**	**0.7877**	0.7811	0.7212	0.7707	0.5508	0.5850	0.5653
SARI	98.1100	**98.9700**	98.2900	97.9009	98.7634	98.0863	**99.1892**	98.2026	**98.3777**
GLEU	**0.3466**	**0.3141**	**0.3419**	0.3163	0.3017	0.3168	0.1756	0.2521	0.2369
WER	**0.7835**	**0.8061**	**0.7876**	0.8110	0.8064	0.8063	0.9374	0.8734	0.8851

The best results for each dataset and metric are highlighted in bold.

**Table 3:** Evaluation metrics for mT5, mT0 and BLOOMZ finetuned for AE with the SQAC dataset.

*Models trained with SQAC (AE)*									
Metric	*mT5*			*mT0*			*BloomZ*		
	SQuAD	SQAC	SQAC + SQuAD	SQuAD	SQAC	SQAC + SQuAD	SQuAD	SQAC	SQAC + SQuAD
Bleu_1	**0.4328**	**0.4157**	**0.4331**	0.4229	0.3961	0.4175	0.2979	0.1796	0.2767
Bleu_2	**0.3487**	**0.3544**	**0.3523**	0.3370	0.3340	0.3359	0.1927	0.1232	0.1794
Bleu_3	**0.3038**	**0.3276**	**0.3107**	0.2924	0.3072	0.2945	0.1532	0.1020	0.1428
Bleu_4	**0.2691**	**0.3093**	**0.2791**	0.2585	0.2887	0.2634	0.1280	0.0883	0.1193
METEOR	**0.2901**	**0.3145**	**0.2948**	0.2841	0.3003	0.2867	0.2276	0.1695	0.2165
ROUGE_L	**0.4729**	**0.5126**	**0.4805**	0.4627	0.4884	0.4666	0.3298	0.2411	0.3128
CIDEr	**2.1617**	**2.6614**	**2.2466**	2.0476	2.3816	2.0955	0.7917	0.6928	0.7819
Cos_sim	0.7608	**0.7617**	**0.7613**	**0.9116**	0.7387	0.7500	0.5672	0.5151	0.5473
SARI	97.8000	98.6900	97.9600	97.8406	98.6082	97.9634	**98.2913**	**99.2272**	**98.4593**
GLEU	**0.3089**	**0.3663**	**0.3191**	0.2995	0.3452	0.3080	0.2225	0.1719	0.2119
WER	0.8988	**0.7408**	**0.8693**	**0.7509**	0.7776	0.8802	1.0182	1.0021	1.0229

The best results for each dataset and metric are highlighted in bold.

**Table 4:** Evaluation metrics for mT5, mT0 and BLOOMZ fine-tuned for AE with the SQAC + SQuAD dataset.

*Models trained with SQAC* + *SQuAD (AE)*									
Metric	*mT5*			*mT0*			*BloomZ*		
	SQuAD	SQAC	SQAC + SQuAD	SQuAD	SQAC	SQAC + SQuAD	SQuAD	SQAC	SQAC + SQuAD
Bleu_1	**0.4369**	**0.4102**	**0.4306**	0.3914	0.3230	0.3819	0.0375	0.0355	0.0324
Bleu_2	**0.3560**	**0.3437**	**0.3527**	0.3151	0.2589	0.3076	0.0206	0.0202	0.0202
Bleu_3	**0.3071**	**0.3123**	**0.3074**	0.2726	0.2332	0.2679	0.0154	0.0156	0.0167
Bleu_4	**0.2661**	**0.2897**	**0.2704**	0.2382	0.2155	0.2357	0.0123	0.0131	0.0151
METEOR	**0.3111**	**0.3104**	**0.3099**	0.2868	0.2749	0.2846	0.1970	0.1915	0.1620
ROUGE_L	**0.5153**	**0.5131**	**0.5124**	0.4928	0.4644	0.4881	0.3407	0.3290	0.2640
CIDEr	**2.4798**	**2.6547**	**2.4924**	2.2391	2.0621	2.2331	0.2377	0.2450	0.2942
Cos_sim	**0.7971**	**0.7603**	**0.7882**	0.7784	0.7319	0.7685	0.6966	0.6436	0.6860
SARI	98.1300	98.7100	98.2500	97.9223	98.6918	98.0496	**98.4814**	**99.1793**	**98.6155**
GLEU	**0.3434**	**0.3575**	**0.3436**	0.3141	0.3100	0.3128	0.2010	0.1658	0.1960
WER	**0.8018**	**0.7593**	**0.7988**	0.8162	0.7916	0.8190	0.8472	0.8908	0.8513

The best results for each dataset and metric are highlighted in bold.

**Table 5:** Evaluation metrics for mT5, mT0 and BLOOMZ finetuned for Question Generation with the SQuAD dataset.

*Models trained with SQuAD (QG)*									
Metric	*mT5*			*mT0*			*BloomZ*		
	SQuAD	SQAC	SQAC + SQuAD	SQuAD	SQAC	SQAC + SQuAD	SQuAD	SQAC	SQAC + SQuAD
Bleu_1	**0.3858**	**0.3294**	**0.3783**	0.3332	0.2681	0.3281	0.2126	0.2431	0.2369
Bleu_2	**0.2868**	**0.2317**	**0.2795**	0.2363	0.1743	0.2300	0.1275	0.1413	0.1412
Bleu_3	**0.2242**	**0.1706**	**0.2171**	0.1788	0.1223	0.1723	0.0806	0.0838	0.0876
Bleu_4	**0.1794**	**0.1308**	**0.1730**	0.1398	0.0897	0.1335	0.0532	0.0528	0.0566
METEOR	**0.2381**	**0.2284**	**0.2368**	0.2159	0.1937	0.2122	0.1611	0.1734	0.1682
ROUGE_L	**0.3694**	**0.3499**	**0.3665**	0.3325	0.2758	0.3219	0.2399	0.2523	0.2506
CIDEr	**1.6472**	**1.4023**	**1.6140**	1.3263	0.9445	1.2671	0.6172	0.6244	0.6285
Cos_sim	**0.6828**	**0.6422**	**0.6768**	0.6503	0.5716	0.6381	0.5704	0.4662	0.5879
SARI	99.7600	99.8600	99.7800	99.7849	**99.8785**	99.7962	**99.8884**	99.8321	**99.8326**
GLEU	**0.2163**	**0.1923**	**0.2131**	0.1897	0.1493	0.1827	0.1190	0.1167	0.1196
WER	0.8526	0.9336	**0.8628**	**0.8521**	1.0045	0.8750	0.9299	**0.8890**	0.9033

The best results for each dataset and metric are highlighted in bold.

**Table 6:** Evaluation metrics for mT5, mT0 and BLOOMZ finetuned for QG with the SQAC dataset.

*Models trained with SQAC (QG)*									
Metric	*mT5*			*mT0*			*BloomZ*		
	SQuAD	SQAC	SQAC + SQuAD	SQuAD	SQAC	SQAC + SQuAD	SQuAD	SQAC	SQAC + SQuAD
Bleu_1	**0.3102**	**0.3727**	**0.3182**	0.2810	0.3363	0.2898	0.2585	0.2585	0.1910
Bleu_2	**0.2228**	**0.2711**	**0.2290**	0.1815	0.2286	0.1894	0.1594	0.1594	0.1032
Bleu_3	**0.1699**	**0.2044**	**0.1743**	0.1264	0.1650	0.1329	0.1022	0.1022	0.0592
Bleu_4	**0.1334**	**0.1588**	**0.1367**	0.0914	0.1230	0.0970	0.0692	0.0692	0.0359
METEOR	**0.2054**	**0.2343**	**0.2091**	0.1872	0.2161	0.1916	0.1825	0.1825	0.1460
ROUGE_L	**0.3201**	**0.3839**	**0.3297**	0.2803	0.3389	0.2909	0.2872	0.2872	0.2132
CIDEr	**1.3087**	**1.6382**	**1.3635**	0.9124	1.2601	0.9689	0.8508	0.8508	0.4644
Cos_sim	0.6340	**0.6521**	**0.6367**	**0.9091**	0.6278	0.6187	0.5490	0.5988	0.5565
SARI	99.7900	99.8800	99.8000	99.8134	99.8766	99.8219	**99.8631**	**99.9027**	**99.8690**
GLEU	**0.1862**	**0.2206**	**0.1906**	0.1487	0.1816	0.1544	0.0924	0.1391	0.0983
WER	0.8454	**0.7949**	**0.8391**	**0.6160**	0.8719	0.9006	0.9324	0.8569	0.9229

The best results for each dataset and metric are highlighted in bold.

**Table 7:** Evaluation metrics for mT5, mT0 and BLOOMZ finetuned for QG with the SQAC + SQuAD dataset.

*Models trained with SQAC* + *SQuAD (QG)*									
Metric	*mT5*			*mT0*			*BloomZ*		
	SQuAD	SQAC	SQAC + SQuAD	SQuAD	SQAC	SQAC + SQuAD	SQuAD	SQAC	SQAC + SQuAD
Bleu_1	**0.3837**	**0.3992**	**0.3856**	0.3379	0.3473	0.3407	0.2173	0.2404	0.2203
Bleu_2	**0.2867**	**0.2967**	**0.2880**	0.2405	0.2422	0.2410	0.1305	0.1472	0.1326
Bleu_3	**0.2252**	**0.2273**	**0.2255**	0.1808	0.1767	0.1806	0.0830	0.0938	0.0843
Bleu_4	**0.1812**	**0.1794**	**0.1811**	0.1398	0.1334	0.1391	0.0550	0.0639	0.0561
METEOR	**0.2382**	**0.2468**	**0.2393**	0.2161	0.2219	0.2174	0.1624	0.1747	0.1640
ROUGE_L	**0.3710**	**0.4036**	**0.3759**	0.3359	0.3528	0.3381	0.2429	0.2674	0.2466
CIDEr	**1.6784**	**1.7813**	**1.6970**	1.3319	1.3455	1.3328	0.6265	0.7694	0.6458
Cos_sim	**0.6826**	**0.6744**	**0.6814**	0.6558	0.6393	0.6533	0.5892	0.5869	0.5889
SARI	99.7600	99.8600	99.7800	99.7856	99.8716	99.7939	**99.8304**	**99.8821**	**99.8382**
GLEU	**0.2190**	**0.2354**	**0.2210**	0.1879	0.1938	0.1885	0.1185	0.1317	0.1202
WER	**0.8424**	**0.7887**	**0.8357**	0.8566	0.8331	0.8558	0.8851	0.8750	0.8838

The best results for each dataset and metric are highlighted in bold.

**Table 8:** Evaluation metrics for mT0 and BLOOMZ base models for the AE task.

*Base models (AE)*						
Metric	*mT0*			*BloomZ*		
	SQuAD	SQAC	SQAC + SQuAD	SQuAD	SQAC	SQAC + SQuAD
Bleu_1	**0.0726**	**0.0727**	**0.0715**	0.0209	0.0347	0.0236
Bleu_2	**0.0472**	**0.0461**	**0.0460**	0.0053	0.0090	0.0062
Bleu_3	**0.0348**	**0.0336**	**0.0336**	0.0017	0.0018	0.0019
Bleu_4	**0.0269**	**0.0259**	**0.0258**	0.0009	0.0000	0.0009
METEOR	**0.0564**	**0.0643**	**0.0573**	0.0189	0.0301	0.0212
ROUGE_L	**0.0799**	**0.0823**	**0.0794**	0.0246	0.0424	0.0279
CIDEr	**0.1586**	**0.1101**	**0.1430**	0.0042	0.0065	0.0056
Cos_sim	**0.1900**	**0.2195**	**0.1943**	0.1157	0.1212	0.1199
SARI	99.8997	99.9347	99.9058	**99.9728**	**99.9659**	**99.9704**
GLEU	**0.0337**	**0.0339**	**0.0329**	0.0062	0.0095	0.0069
WER	**2.3521**	**2.5636**	**2.3998**	3.0549	3.0763	3.0935

The best results for each dataset and metric are highlighted in bold.

**Table 9:** Evaluation metrics for mT0 and BLOOMZ base models for the QG task.

*Base models (QG)*						
Metric	*mT0*			*BloomZ*		
	SQuAD	SQAC	SQAC + SQuAD	SQuAD	SQAC	SQAC + SQuAD
Bleu_1	**0.2038**	**0.2065**	**0.2054**	0.0820	0.1459	0.1134
Bleu_2	**0.1262**	**0.1232**	**0.1268**	0.0421	0.0776	0.0581
Bleu_3	**0.0878**	**0.0803**	**0.0872**	0.0237	0.0448	0.0322
Bleu_4	**0.0650**	**0.0553**	**0.0637**	0.0144	0.0286	0.0194
METEOR	**0.1582**	**0.1585**	**0.1584**	0.1020	0.1306	0.1128
ROUGE_L	**0.2314**	**0.2129**	**0.2291**	0.1314	0.1699	0.1491
CIDEr	**0.6811**	**0.6525**	**0.6852**	0.2217	0.3639	0.2742
Cos_sim	**0.5496**	**0.5010**	**0.5426**	0.2496	0.2393	0.2699
SARI	99.8418	99.9076	99.8537	**99.9169**	**99.9256**	**99.9047**
GLEU	**0.1224**	**0.1157**	**0.1214**	0.0632	0.0841	0.0711
WER	**0.8940**	0.9820	**0.9063**	0.9338	**0.9477**	0.9397

The best results for each dataset and metric are highlighted in bold.

Regarding the results of the models fine-tuned with SQuAD for Answer Extraction shown in Table 2, mT5 outperforms the other ones in all the evaluation metrics except in SARI, where BLOOMZ achieves better results for the SQuAD and SQAC + SQuAD datasets.

According to the results shown in Table 3 about the models fine-tuned with SQAC for Answer Extraction, mT5 achieves better results for each metric except for WER, cosine similarity and SARI. Indeed, BLOOMZ obtains better results for SARI while mT0 obtains the best cosine similarity and WER values for the dataset SQuAD.

Concerning the results about the models fine-tuned with SQAC + SQuAD for Answer Extraction, shown in Table 4, mT5 outperforms the results obtained except for SARI, where BLOOMZ outperforms the other results.

Analyzing the scores of the first three tables, the models fine-tuned with the union of SQAC and SQuAD datasets achieve, in general, better results for the evaluation metrics computed.

Focusing on the results about the performance of the three models fine-tuned with SQuAD in Question Generation (Table 5), mT5 achieves the best results for all the metrics except for SARI and WER. The best results for the SARI metrics are achieved by mT0 for SQAC and by BLOOMZ for SQuAD and SQAC + SQuAD. The best results for the WER metric are obtained by mT0 for SQuAD, by BLOOMZ for SQAC and by mT5 for SQAC + SQuAD.

According to the results obtained by models fine-tuned with SQAC for Question Generation (Table 6), mT5 achieves the best results except for WER and SARI. Concretely, BLOOMZ obtains better results for the SARI metric and mT0 outperforms the results of cosine similarity and WER in SQuAD.

Regarding the models fine-tuned with SQAC and SQuAD for Question Generation (Table 7), mT5 also outperforms the other models in all metrics except SARI, where BLOOMZ achieves better results.

Analyzing the results obtained (Tables 5, 6, 7), the models fine-tuned with SQAC + SQuAD achieve, in general, better results for the evaluation metrics computed.

Finally, regarding the results obtained from the base models (not fine-tuned), applying a zero-shot approach, for the task of AE (Table 8) and QG (Table 9), mT0 achieves better results in both tasks except for the metrics WER and SARI, where BLOOMZ outperforms the results. Specifically, BLOOMZ achieves better results for SARI in all datasets in both tasks, for WER in all datasets for AE and in SQuAD and SQAC + SQuAD for QG.

Once all the evaluation metrics are computed, some results must be highlighted.

### A. MT5 models

Regardless of the dataset used, mT5 models achieve, in general, the best results for every evaluation metric except SARI, where the other models sometimes achieve better results. As an example, the best results according to the BLEU4 metrics for QG and AE are obtained with these models.

Focusing on the values obtained, the best mT5 model is the one fine-tuned with the union of SQAC and SQuAD datasets, with very little difference between the one fine-tuned only with SQuAD. Even though the results obtained with the union dataset are the best ones in general, it is important to stand out that some of the best results are obtained from the model fine-tuned only with SQAC (e.g., BLEU4 for AE).

### B. MT0 models

Although they are not the best ones, results demonstrate that scores of mT0 models are near to the mT5 ones. In a similar way to mT5, the values obtained show a better performance of the model fine-tuned with the union dataset, followed by the one fine-tuned with SQuAD. However, the model fine-tuned with SQAC achieved the highest cosine similarity of the research study for the dataset SQuAD. Another important fact is that, focusing only on the results obtained by the mT0 models, the one fine-tuned with SQAC achieves the best results for BLEU1-4 and METEOR metrics in the AE task. Additionally, the one fine-tuned with the union dataset obtains the best results for the QG task.

### C. Bloomz models

Even though these models provide the worst results in our research study, there is an important detail that must be mentioned. Focusing on the results, a huge difference exists between the ones obtained from the model fine-tuned with SQuAD and the ones from the model fine-tuned with the union dataset. Please note that the first ones are the best for BLOOMZ models, while the others are the worst.

### D. Base models

According to the results shown, the zero-shot technique with mT0 is more effective for the AE and QG tasks. However, their performance is lower in comparison with the previous models, which are fine-tuned for the tasks at hand, as it is expected^61^.

## Discussion and conclusion

Answer Extraction and Question generation are ones of the most difficult NLP tasks^71^. Furthermore, these problems become even more challenging when languages other than English are used to address them. As a result, in other languages, such as Spanish, there is a huge lack of research about these problems. The appearance of multilingual models and new Spanish datasets is helping the development on NLP research regarding AE and QG.

In this study, we have taken the research made by Ricardo-Torrealba^41^ and Chan and Fan^17^ as baselines for our work. Following their studies, we have applied them to Spanish by fine-tuning three multilingual models (mT5-base, mT0-base and BLOOMZ-560 M) with three different datasets in Spanish (SQAC, SQuAD, SQAC + SQuAD). Computing a wide range of metrics, their performance has been evaluated on the AE and QG tasks.

Our results demonstrate that, following an answer-aware QG technique with the mT5 model finetuned with SQAC + SQuAD, it is possible to outperform certain prior research for QG in English. For example, regarding the results provided in ^20,25,26^ in QG for BLEU4 and METEOR on the SQuAD dataset, there is a noticeable improvement. Moreover, our approach achieves slightly better results for BLEU4 and METEOR than the ones obtained by Liu et al.^23^. Additionally, we achieved better results for BLEU2-4 than the work proposed by Sun et al.^39^ and improved BLEU4 results for the SQuAD dataset in^28^. Finally, comparing the results obtained by Wang et al.^18^ in QG for BLEU3-4 and METEOR on the whole SQuAD dataset, we achieve some improvement. In addition, our study outperforms significantly the results presented by Ushio et al.^53^ for the BLEU4, ROUGE-L and METEOR metrics obtained for QG in Spanish using mT5. This study, which is the only one that provides results for Spanish QG among all the related research found, evaluates different language models on QG-Bench dataset, a unified collection of datasets with the same format in which SQuAD^57^ is included. The improvements achieved by our approach compared to all these studies are because of two main reasons: (1) effectiveness of data preprocessing that avoid raising the complexity of the system. Unlike other strategies that apply complex techniques such as masking the answer from the passage^25^, matching strategies between passage and answers^20,26^ or hybrid answer-focused and position-aware models^39^, our approach is able to stand out the relationship between the passage and its associated answer without increasing the complexity of the system. Thanks to this preprocessing technique, the increase of complexity in the pipeline is avoided, making easier the understanding of this relationship to the model. The second reason is (2) the potential performance of the multilingual models used. Even though in this study it was not possible to use bigger models due to hardware limitations, the architectures of these models and their huge pretraining make it possible to outperform more sophisticated architectures such as the ones proposed by S. Wang et al.^18^, which uses knowledge graphs and divides the task of question generation into two steps, query representation and query-based question generation, or Liu et al.^23^, which builds the questions by identifying where each word in the question should came from a vocabulary or copied from the input text.

However, we are not able to outperform all the results found in literature. Comparing to our baselines, we almost reach Ricardo-Torrealba et al.^41^ BLEU4 (21.32), METEOR (27.09) and ROUGE-L (43.59) metrics obtained for English QG, which uses T5 fine-tuned models by Patil^72^. Moreover, our results are near the ones obtained by Chan and Fan^17^ using BERT for English QG in BLEU4 (20.33), METEOR (23.88) and ROUGE-L (48.23). Furthermore, the approach proposed by Sasazawa et al.^24^ clearly improved our results with the incorporation of an interrogative phrase at the end of the passage. Also, the technique of selecting the best answer using a confidence score for the AE task and the QG technique proposed by Back et al.^40^ improved our results. Finally, the model proposed by Murakhovs’ka et al.^22^, employing a total of nine English datasets, obtained the best results of all previous research without using the interrogative phrases technique^24^, raising awareness of the importance of the amount of data used for training. The improvement achieved by these works compared to our results could be achieved, in some way, due to the use of the English language, having more resources and prior research than in Spanish (e.g. Murakhovs’ka et al.^22^ used nine available datasets for QA in English). Nevertheless, approaches such as the ones followed by Sasazawa et al.^24^, which used a different input format using interrogative sentences, might facilitate the question generation task to the model, resulting in obtaining better results. Additionally, the idea proposed by Back et al.^40^ of obtaining several answers, ranking them and choosing the best ones, could bring a certain error range that is later corrected by choosing the best answer that has been generated, thus improving the results. Table 10 compares our results to those achieved by the studies that define our baseline and other related work.

**Table 10:** Comparison with existing question generation research.

Research study	Model	Dataset (lang)	B1	B2	B3	B4	METEOR	ROUGE-L
Chan and Fan^17^	BERT-HLSQG Paragraph level	SQuAD (en)	49.73	34.60	26.13	20.33	23.88	48.23
Wang et al.^18^	PathQG-V	SQuAD (en)	50.14	32.25	22.48	15.98	18.85	43.46
Song et al.^20^	M2S + cp	SQuAD (en)	–	–	–	13.98	18.77	42.72
Murakhovs'ka et al.^22^	mixQG	SQuAD (en)	–	–	–	25.42	45.75	51.85
Liu et al.^23^	CGC-QG	SQuAD (en)	46.58	30.90	22.82	17.55	21.24	44.53
Sasazawa et al.^24^	Ass2s Answer excluding interrogative	SQuAD (en)	–	–	–	13.70	17.70	40.80
Sasazawa et al.^24^	Seq2Seq Answer excluding interrogative	SQuAD (en)	–	–	–	17.90	20.40	45.20
Sasazawa et al.^24^	Ass2s normal evaluation	SQuAD (en)	–	–	–	21.60	23.00	51.20
Sasazawa et al.^24^	Seq2Seq normal evaluation	SQuAD (en)	–	–	–	26.40	26.20	55.90
Kim et al.^25^	ASs2s	SQuAD (en)	–	–	–	16.20	19.92	43.96
Ma et al.^26^	Combined Model	SQuAD (en)	44.71	29.89	21.77	16.32	20.84	44.79
Sun et al.^28^	–	SQuAD (en)	47.28	31.35	23.02	17.52	–	46.78
Sun et al.^39^	Hybrid model	SQuAD (en)	43.02	28.14	20.51	15.64	–	–
Back et al.^40^	ProphetNet + ASGen	SQuAD (en)	–	–	–	24.40	26.70	52.80
Ricardo-Torrealba^41^	t5-base-qg-hl	SQuAD (en)	–	–	–	21.32	27.09	43.59
Ushio et al.^53^	mT5 Base Spanish	QG-Bench (es)	–	–	–	10.15	23.43	25.45
*QG mt5 base SQAC* + *SQuAD*	*(our model)*	*SQuAD (es)*	*38.37*	*28.67*	*22.52*	*18.12*	*23.82*	*37.10*

BLEU1-4, METEOR and ROUGE-L metrics.

Once the results are analyzed, five important facts can be highlighted. (1) The importance of high-quality Spanish datasets. (2) The importance of the amount of data used for training. (3) The significance of the finetuning process for AE and QG in multitask models. (4) The bias of the dataset towards a specific task. (5) The influence of multitask finetuning in the future use. These ideas are explained below.

Firstly, it has been shown that a high-quality dataset such as SQAC, built directly in Spanish without automatic translation, can significantly improve the performance of the models. Furthermore, it makes this improvement with a very low proportion of data compared to the SQuAD dataset. As a result, there is an important need to build high-quality datasets directly in Spanish, without automatic translation, to improve the performance of generative models.

Secondly, the results have shown that the greater the availability of data, the better behavior of the models. In general, the models trained with the combination of SQAC and SQuAD provide a better performance. However, this does not mean that it is better to create huge datasets leaving aside the quality of data. Indeed, the real objective should be the creation of bigger Spanish datasets with quality data focused on the AE and QG tasks.

Thirdly, regarding the results obtained for the base models, they are significantly worse than the others obtained by applying finetuning. Even though mT0 and BLOOMZ models were pretrained for multiple tasks and the same format was followed, it has been shown that a more precise training is needed to achieve better results. However, once finetuned these models, they achieve worse results than mT5, which is not a multitask model. Therefore, for the QG and AE tasks, the multitask training carried out seems to be insufficient. Please note that the size of the model takes an important role here, so zero-shot performance of bigger models is expected to be better.

Furthermore, when analyzing the outcome obtained, the SQuAD dataset provides more balanced metrics between the AE and QG tasks, while the SQAC results show off more correctness in the AE task. Consequently, it can be deduced that the nature of the dataset plays a very important role in the performance of each task. On the one hand, on average, SQAC has a longer passage length, which seems to improve the understanding of the model, obtaining more precisely the answers. On the other hand, SQuAD contains shorter questions than SQAC, being easier to obtain better metrics. As a result, longer passages improve the performance on AE. However, the performance of QG is biased by the length of the question because, since metrics compare n-grams, the longer the question is, the more difficult it will be to obtain it.

Finally, as shown with the BLOOMZ model, the results obtained are highly biased by the finetuning given to this model. Even though BLOOMZ is a multilingual model, it has been mostly pretrained with English datasets for the tasks of multiple choice and extractive question answering such as SQuAD. Hence, the results for Spanish datasets that were not used during the pretraining phase are very poor (e.g., SQAC). However, better results are obtained for the Spanish version of SQuAD, since the English version has been used in the pretraining process.

In conclusion, we have evaluated and compared through automatic evaluation metrics the performance of three multilingual models in the AE and QG tasks, using three different Question Answering datasets. Our results show that the best approach to solve the AE and QG problems in Spanish is the mT5 model fine-tuned with the union of SQAC and SQuAD, being even capable of outperforming some of the BLEU and METEOR metrics found on previous research in English. However, although we improve the results of some previous research studies, some other prior research studies in English achieve a better performance, standing out the lack of research for NLP in Spanish. Regarding the ROUGE-L metric, our models obtain worse results but, as it has been explained before, it is not a good discriminator for QG. Indeed, it is better for evaluating AE performance, where we obtained better results for this metric. However, there is no literature found to compare our results for this task. Consequently, we encourage future research to evaluate AE performance. Additionally, we propose the results of this study, including less common metrics but more adequate ones, such as cosine similarity, as a benchmark for future work within the AE and QG problems in Spanish.

### Supplementary Information

Supplementary Information.

## Data Availability

The datasets generated during and/or analysed during the current study are available from the corresponding author on reasonable request.
