# Supplementary material for: Evaluating the performance of multilingual models in answer extraction and question generation
Source: Sci Rep. 2024 Jul 5;14:15477. doi: 10.1038/s41598-024-66472-5 (PMC11226668; doi:10.1038/s41598-024-66472-5)
Supplement: Supplementary file 1 — Supplementary Information. [file 41598_2024_66472_MOESM1_ESM.docx]

**APPENDIX A**

**DATA PREPROCESSING ALGORITHMS**

Below are the different algorithms that have been used for data preprocessing in this study. Please note that they are written in pseudocode.

**
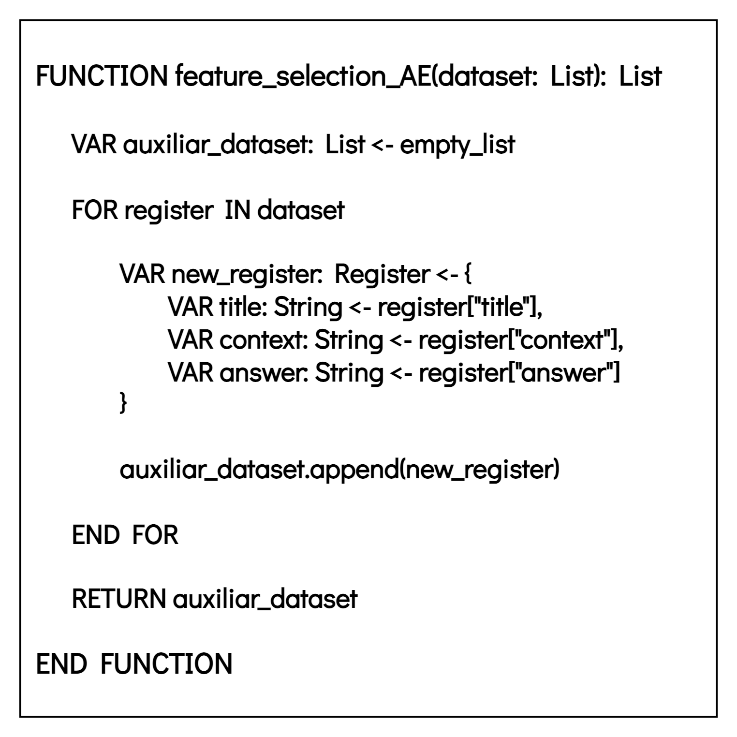
**

**Supplementary Algorithm S1.** **Function used to choose the relevant features to train the models. For this task, Answer Extraction, the title, context, and answer are selected.**


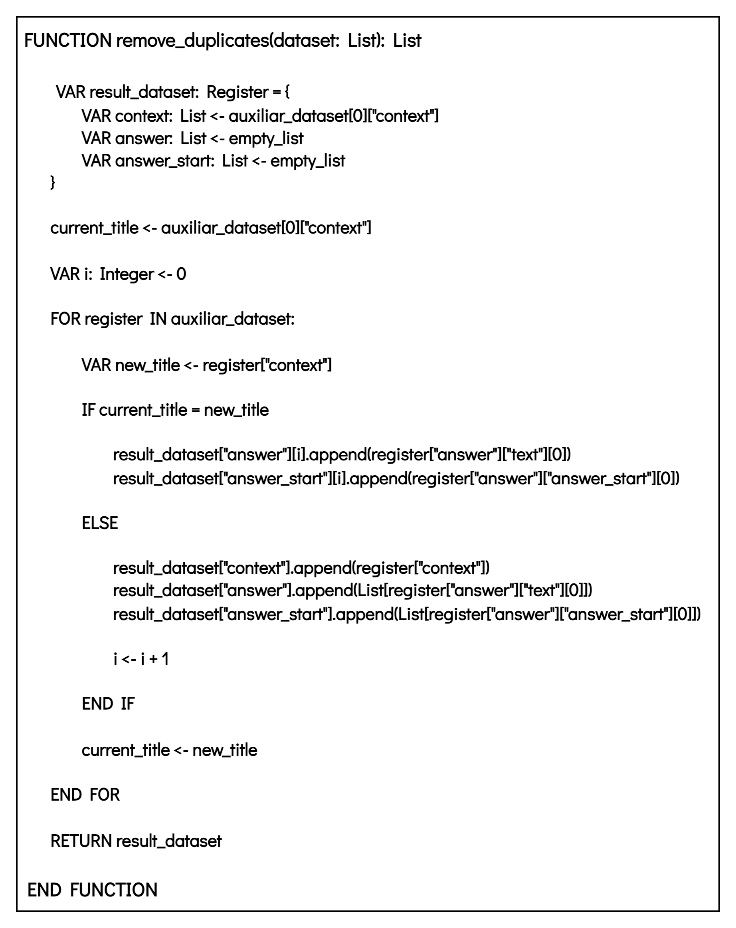


**Supplementary Algorithm S2.** **This algorithm leverages the title of each register to gather every register with the same title in one single structure. Thereby, those duplicated contexts are removed.**


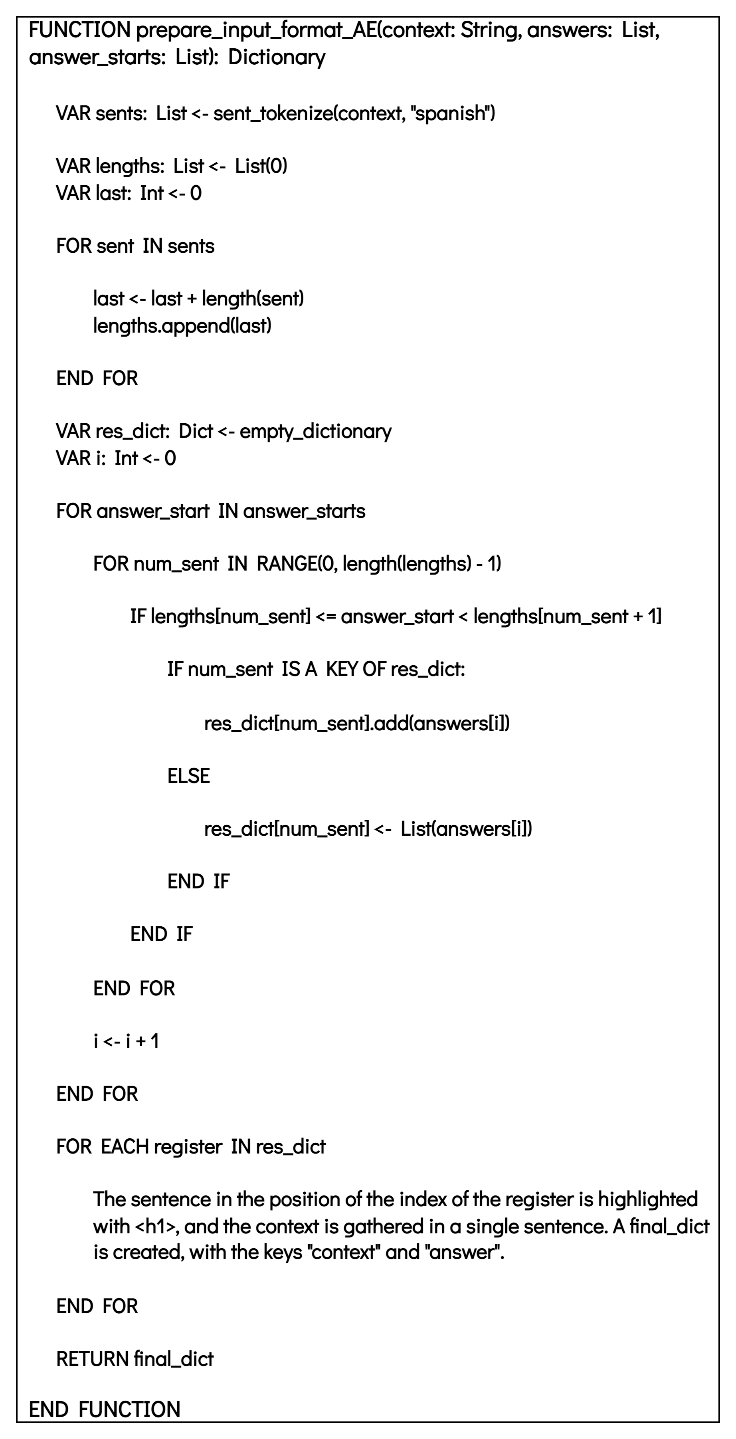


**Supplementary Algorithm S3.** **This function divides each context into its respective sentences using *sent_tokenize*. Then, every answer of each register is classified for every sentence, and those sentences who have answers among their text are highlighted with the special token <h1>**


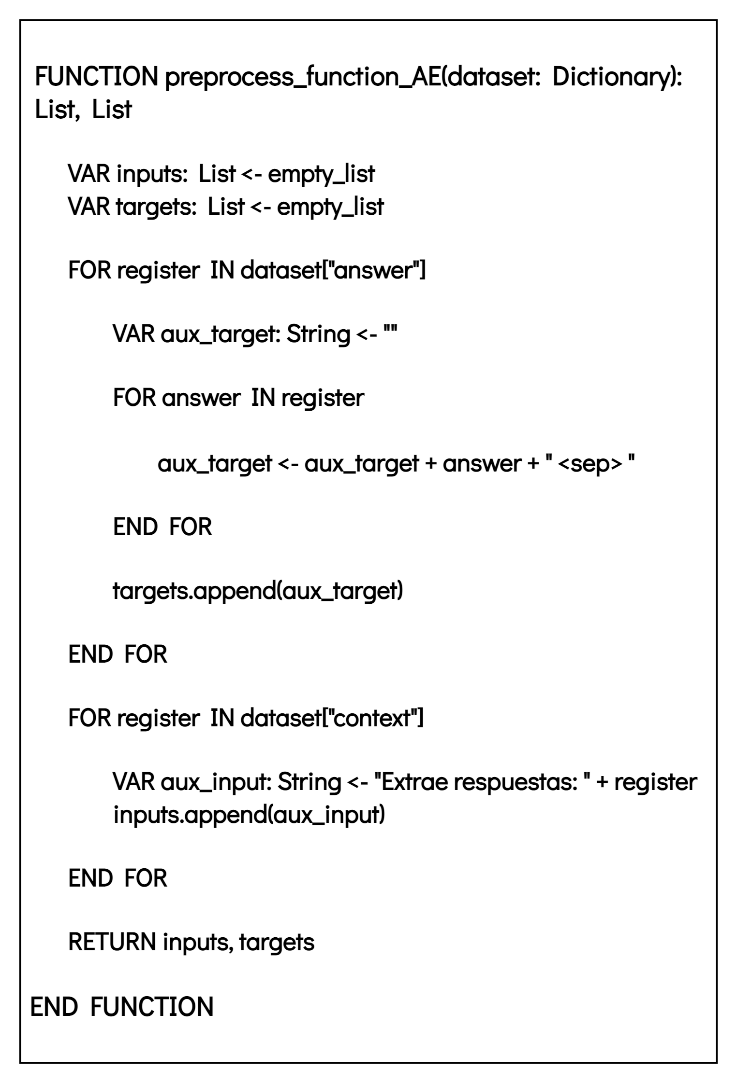


**Supplementary Algorithm S4.** **Function used to add the prompt and prepare the input to train the mT5 model for the Answer Extraction task. In the first loop, the answers of each register are gathered in one String but separated by the special token “<sep>”. In the second one, the prompt is added to each context in the dataset. The translation of the prompt “Extrae respuestas:” into English is: “Extract answers:”.**


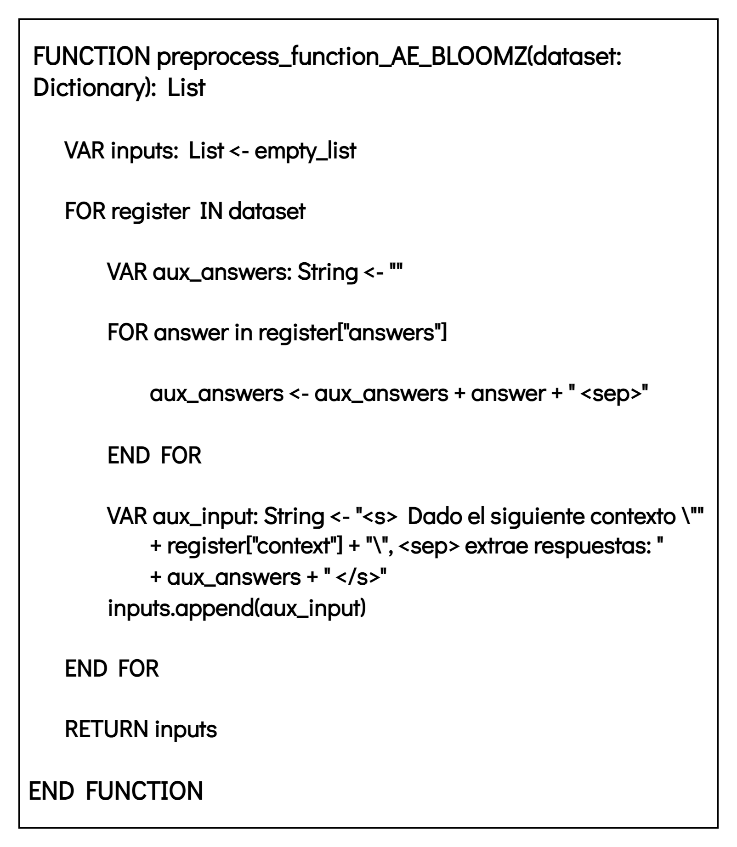


**Supplementary Algorithm S5.** **Function used to add the prompt and prepare the input to train the BLOOMZ model for the Answer Extraction task. In the first loop, all the registers of the dataset are taken one by one, gathering all the correspondent answers for each register in the second loop. The input is written in one String and added to the inputs list.The translation of the prompt “Dado el siguiente contexto […], <sep> extrae respuestas […]” into English is: “Given the following context […], <sep> extract answers […]”.**


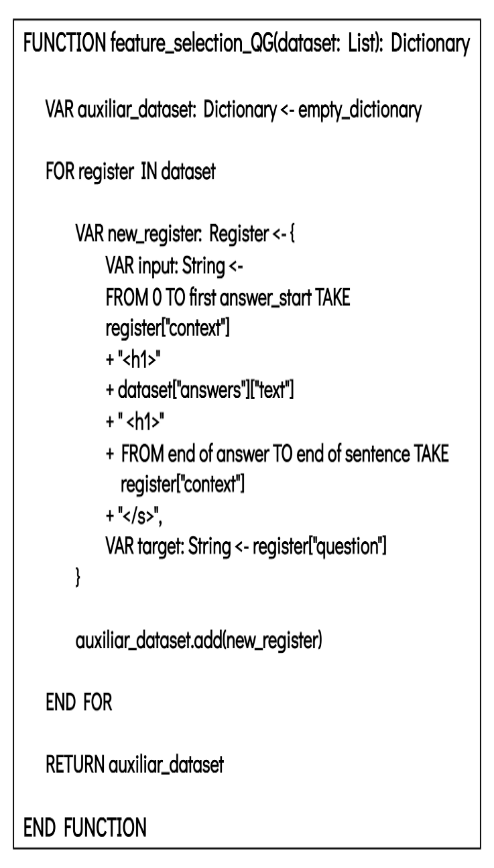


**Supplementary Algorithm S6.** **Function used to choose the relevant features to train the mT5 model. For this task (Question Generation), the algorithm extracts, for each register, its context, answer, and question. Using these attributes, the answer is highlighted inside the context with <h1>, then, this modified context is stored as the model input, and the question is stored as the target that the model will try to infer.**


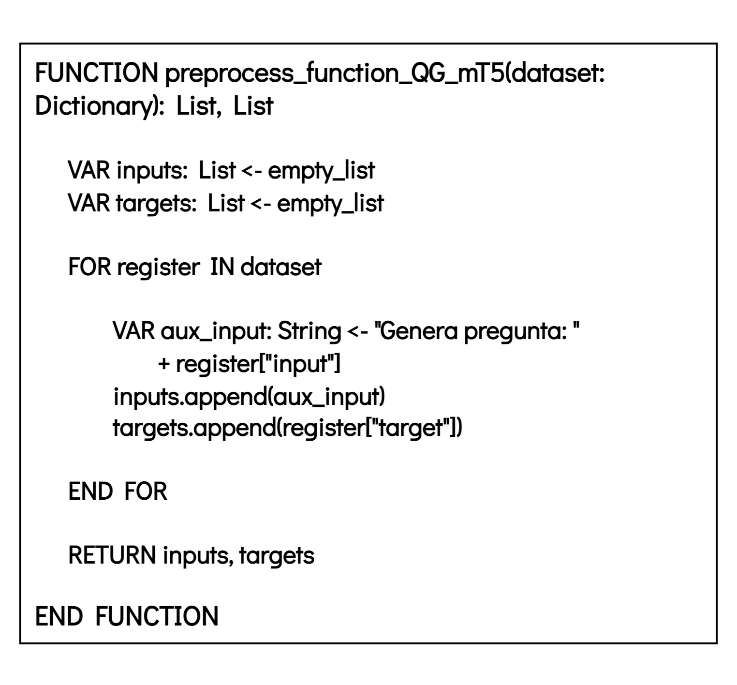


**Supplementary Algorithm S7.** **Function used to add the prompt and prepare the input to train the mT5 model for the Question Generation task. The translation of the prompt “Genera pregunta:” into English is: “Generate question:”.**


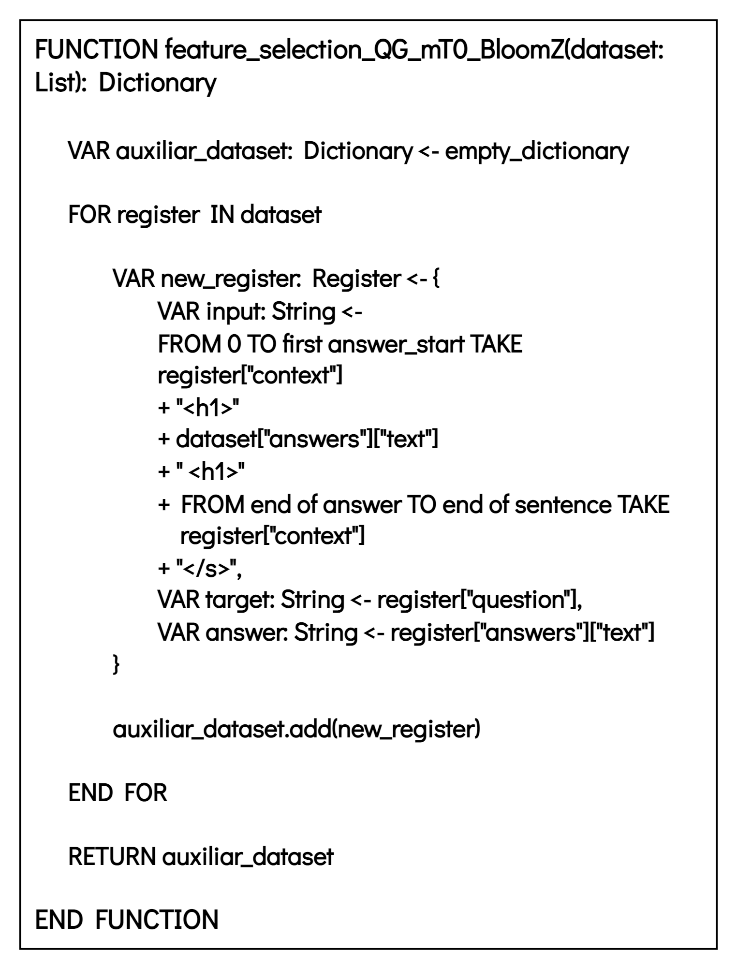


**Supplementary Algorithm S8.**  **Function used to choose the relevant features to train the mT0 and BLOOMZ models. For this task (Question Generation), the algorithm extracts, for each register, its context, answer, and question. Using these attributes, the answer is highlighted inside the context with <h1>, then, this modified context is stored as the model input, and the question is stored as the target that the model will try to infer.**

**Please note that, unlike in Algorithm 6, the answer is also selected as an attribute of the new register, since it is later used in the preprocess function.**


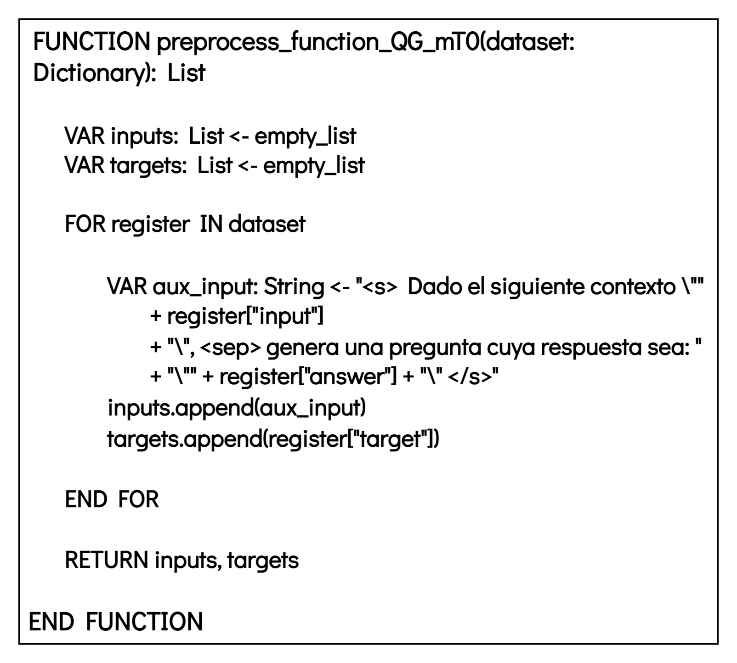


**Supplementary Algorithm S9.**  **Function used to add the prompt and prepare the input to train the mT0 model for the Question Generation task. The translation of the prompt “Dado el siguiente contexto […], <sep> genera una pregunta cuya respuesta sea […]” into English is: “Given the following context […], <sep> generate a question whose answer would be […]”.**


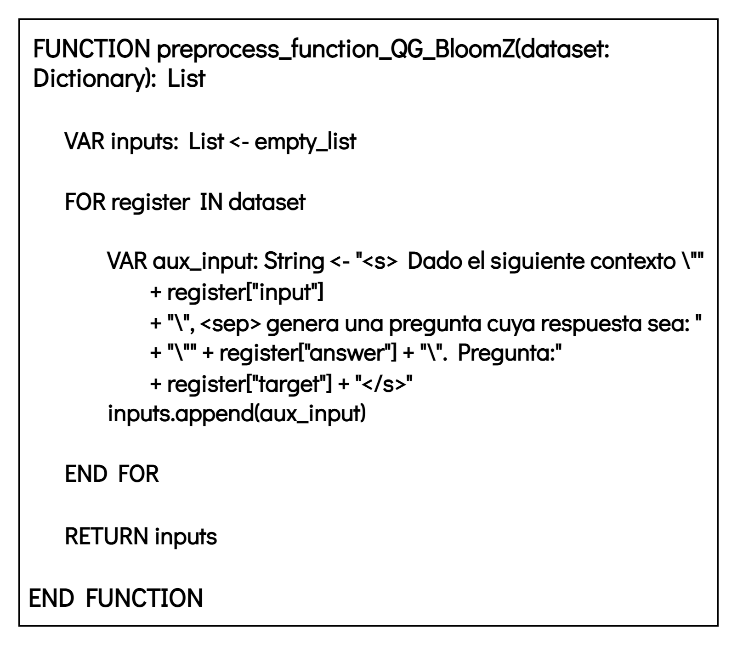


**Supplementary Algorithm S10.**  **Function used to add the prompt and prepare the input to train the BLOOMZ model for the Question Generation task. The translation of the prompt “Dado el siguiente contexto […], <sep> genera una pregunta cuya respuesta sea […]” into English is: “Given the following context […], <sep> generate a question whose answer would be […]”.**
